# National multi-stakeholder meetings: a tool to support development of integrated policies and practices for testing and prevention of HIV, viral hepatitis, TB and STIs

**DOI:** 10.1186/s12879-021-06492-y

**Published:** 2021-09-13

**Authors:** Daniel Simões, Raimonda Matulionyté, Loreta Stoniene, Piotr Wysocki, Justyna Kowalska, Nadia Gasbarrini, Lella Cosmaro, Tatjana Nemeth Blažić, Arian Dišković, Zoran Dominković, Iva Jovovic, Ilona Razmiene, Manuel Maffeo, Stine F. Jakobsen

**Affiliations:** 1 EPIUnit - Instituto de Saúde Pública, University of Porto, Porto, Portugal; 2 Department of Infectious Diseases and Dermatovenerology, Faculty of Medicine, Vilnius University, Vilnius University Hospital Santaros Klinikos, Vilnius, Lithuania; 3 Republican Centre for Addictive Disorders, Kaunas, Lithuania; 4 National AIDS Centre Agency of the Ministry of Health, Warsaw, Poland; 5 Medical University of Warsaw, Warsaw, Poland; 6 Fondazione Villa Maraini Onlus, Rome, Italy; 7 Fondazione LILA Milano – Italian League for Fighting AIDS, Milano, Italy; 8 Croatian Institute of Public Health, Zagreb, Croatia; 9 Croatian Association for HIV and Viral Hepatitis (HUHIV), Zagreb, Croatia; 10 Iskorak (Sexual and Minorities Rights Center), Zagreb, Croatia; 11 Life Quality Improvement Association (Flight), Zagreb, Croatia; 12 National Public Health Surveillance Laboratory, Vilnius, Lithuania; 13 Arcigay Associazione LGBTI Italiana, Rome, Italy; 14 CHIP- Centre of Excellence for Health, Immunity and Infections, Rigshospitalet, University of Copenhagen, Blegdamsvej 9, 2100 Copenhagen Ø, Denmark

**Keywords:** Integrating services, HIV, Viral hepatitis, Tuberculosis, Sexually transmitted infections

## Abstract

**Background:**

Country level policies and practices of testing and care for HIV, viral hepatitis and sexually transmitted infections are lagging behind European recommendations on integration across diseases. Building on previous experiences and evidence, the INTEGRATE Joint Action arranged four national stakeholder meetings. The aim was to foster cross-disciplinary and cross-disease collaborations at national level as a vehicle for strengthened integration of testing and care services. This article presents the methodology and discusses main outcomes and recommendations of these meetings.

**Methods:**

Local partners in Croatia, Italy, Lithuania and Poland oversaw the planning, agenda development and identification of key persons to invite to ensure that meetings addressed main challenges and issues of the respective countries. Invited national stakeholders represented policy and public health institutions, clinical settings, testing sites and community organisations. National experts and experts from other European countries were invited as speakers and facilitators. Main topic discussed was how to increase integration across HIV, viral hepatitis and sexually transmitted infections in testing and care policies and practice; tuberculosis was also addressed in Lithuania and Italy.

**Results:**

The agendas reflected national contexts and the meetings provided a forum to engage stakeholders knowledgeable of the national prevention, testing and care systems in interaction with international experts who shared experiences of the steps needed to achieve integration in policies and practice. The evaluations showed that participants found meetings relevant, important and beneficial for furthering integration. Of the respondents 78% agreed or strongly agreed that there was a good representation of relevant national stakeholders, and 78% that decision/action points were made on how to move the agenda forward. The importance of securing participation from high level national policy makers was highlighted. Outcomes were nationally tailored recommendations on integrated policies and strategies, diversification of testing strategies, stigma and discrimination, key populations, cost effectiveness, surveillance and funding.

**Conclusions:**

Shifting from single to multi-disease approaches require collaboration among a broad range of actors and national multi-stakeholder meetings have proven excellent to kick-start this. Face-to-face meetings of key stakeholders represent a unique opportunity to share cross-sectoral perspectives and experiences, identify gaps in national policies and practices and agree on required next steps.

**Supplementary Information:**

The online version contains supplementary material available at 10.1186/s12879-021-06492-y.

## Background

International and European organizations recommend integrating testing and care services for HIV, viral hepatitis and sexually transmitted infections (STIs), because these diseases have common modes of transmission, with significant overlaps in affected population groups and high levels of co-infection [1]. In most European countries existing health care systems, testing sites and community infrastructure provide a strong basis for the implementation of multi-disease policies and practices, with a potential very high impact for individual and public health [1]. Existing structures attended by key populations, especially in urban environments, are evident places to offer testing for multiple infections, and increased investments in targeted interventions and strong focus on facilitating linkage to care and treatment for HIV, viral hepatitis and STI, may rapidly lead to significant improvements in the continuum of care for all the infections.

However, while inventories exist of success models and best-practices, which can be replicated and adapted to local contexts [1, 2], not all European countries have implemented European and international guidance and some health services continue to operate with disease-specific specialties, mandates, budgets and information systems. This results in siloed working and limited collaboration, which in turn means that opportunities to identify common goals, maximise service efficiency and accessibility, and share resources and rewards (i.e. the benefits of integration) are missed [3].

At the national level, cross-disciplinary and cross-disease-area collaboration is a good starting point for integrating service delivery of testing, prevention and linkage-to-care across diseases. Integration is here defined as multi-disease funding schemes, service delivery and informational systems for HIV, viral hepatitis, TB and STI [4]. 'The authors selected multi-stakeholder meetings as an appropriate method to initiate collaboration because a broad array of national key stakeholders is brought together and provided a forum for discussion and reaching a common understanding of the required actions and next steps. This article presents the results of four such meetings held in 2019 in the capitals of Croatia, Italy, Lithuania and Poland arranged by the INTEGRATE Joint Action [5]. The aim of these meetings was to foster cross-disciplinary and cross-disease collaborations at national level as a vehicle for strengthened integration of testing and care services. The meetings drew on experiences and evidence from the project Optimising Testing and Linkage to Care for HIV across Europe [6–11], and were arranged in collaboration with EuroTEST [12] and European Testing Week [13]. The article focuses on the meetings as a method, and the participant evaluations and the main outcomes and recommendations identified for each country as the results.

## Methods

**Fig. 1:**
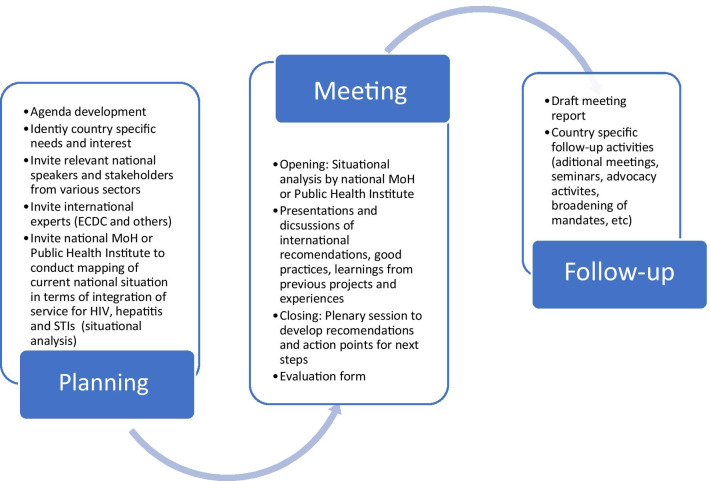
The multi-stakeholder meeting approach

The process of organizing the meetings was led by the INTEGRATE partners from the respective countries and the coordinator CHIP, Rigshospitalet, Denmark [14]. The process of setting up the meetings, the meeting format and follow up-steps are shown in Fig. 1. The first step was agenda development; identification of specific topics of interest and relevant speakers were chosen on the basis that they would facilitate the sharing of good practices and learnings from previous successful national and international projects or experiences. All meetings had participation of a representative from the European Center for Disease Prevention and Control (ECDC) presenting the main points from the 2018 European guidance on integrated testing for HIV and viral hepatitis [1], relevant INTEGRATE partners from other countries and international topic experts.

A uniform structure was developed for meeting openings and closures. All meetings would open with a presentation by a national Ministry of Health (MoH) or Public Health Institute representative, presenting a situational analysis mapping the current national situation considering the aforementioned ECDC guidance (see Additional file 1: template in Annex 1). The situational analyses drew on country data and responses reported to Dublin Declaration Monitoring Questionnaire [15]. Meetings would close with a plenary session where participants formulated recommendations and action points necessary to implement changes towards more integrated testing and care approaches. The rest of the meetings would focus on country specific topics identified by the national organizers in consultation with key national stakeholders. The second step was to identify and invite key stakeholders which would enable national discussion of multi-disease approaches, including scientists, clinicians, political decision makers and advisors, and civil society experts. Simultaneous translations would be provided of the presentations and discussions from English to national language and vice versa. Finally, an evaluation form was developed to elicit uniform feedback from participants across meetings (Additional file 2: Annex 2).

As an add-on to the meetings in Lithuania and Croatia a training session was planned for the day after the main-meeting closure. As for the multi-stakeholder meetings, the national organizers identified topics of relevance, trainers and participants. Both trainings drew on the ESTICOM training programme and focused on service provision, staff attitudes and stigma and discrimination as barriers for testing and linkage to care [16]. In Lithuania the trainers were national experts trained by ESTICOM, while in Croatian they were international ESTCOM trainers. The trainers recommended that the training sessions were held face-to-face in order to secure the best outcome in terms of staffs’ reflection on their own attitudes. Translation was provided from English to national language and vice versa as required. To evaluate the trainings the core indicators in the evaluation form in Additional file 2: Annex 2 were used with slightly adapted wording.

## Results

An overall description of the four national stakeholder meetings is provided in Table 1, including the key topics addressed, profile of presenters, number of meeting participants (35–57) and the evaluation survey response rate (32–47%).

**Table 1:** Overall description of national stakeholder meetings and trainings

Meetings	Place and date	Key topics	Profile of presenters	# of Participants	Evaluation response rate (%)
Croatia	Zagreb, 11 April 2019	Gaps and barriers to testing at individual, provider and institutional levels; How to improve testing strategies including integrated testing and de-medicalisation of testing and linkage to care	National public health, clinical and community experts; ECDC representative; International community testing experts	57	27 (47%)
Lithuania	Vilnius, 3 June 2019	Integration of TB testing with HIV, viral hepatitis and STI services; Current testing policy and practices, cost-effectiveness of different testing strategies; How to improve linkage to care, and existing gaps and barriers	National public health, clinical and community experts; ECDC representative; International experts in modelling, IC Condition-guided HIV and public health	35	16 (46%)
Poland	Warsaw, 18 June 2019	How to implement integrated testing and enhance linkage to care of people newly diagnosed with HIV; Good practice and barriers for integrated testing in health care and community settings; Barriers to the integration of data; Legal aspects of integrated testing in Poland; Best European practice in overcoming legal barriers for integrated testing	National public health, clinical and community experts; ECDC representative; Legal expert	41	19 (46%)
Italy	Rome, 15 November 2019	Towards a better integration of early diagnosis and linkage to care for HIV, viral hepatitis, TB and STIs; Inclusion of data from community testing sites into the national surveillance system; Partner Notification, HIV self-testing policies and ICT tools as additional resources for combination prevention	National public health, clinical and community experts; ECDC representative; Legal expert on Self-Testing	39	17 (44%)

Trainings	Place and Date	Key topics	Profile of Presenters	# of Participants	Evaluation response rate (%)
Croatia	Zagreb, 12 April 2019	Country case examples of integrated testing services in community and health care settings; Training in cultural competency to challenge stigma and discrimination	International community testing experts; National clinical experts; International trainer and facilitator	30	11 (37%)
Lithuania	Vilnius, 4 June 2019	Tools for staff to conduct provider-initiated testing, address the challenges of linkage to care and strategies to motivate clients to enter treatment	National public health, clinical and community experts; International expert in IC Condition-guided HIV; National trainer and facilitator	25	12 (48%)

A broad array of key stakeholders participated, though with important variations across the four meetings as shown in Table 2. Roughly, across meetings (N = 79) a third of participants worked in community-based organisations, NGOs or low-threshold services (35%), a third in hospitals, clinics or laboratories (31%) and a third in government or public health institutions (28%).

**Table 2:** Participants' Profile: Respondents’ place of work

Meetings	Community-based organisations/NGOs/ Low-threshold	Hospitals, clinics or laboratories	Government institutions	Public health institutions	Research or academia	Other
Croatia (N = 27)	12 (44%)	2 (7%)	1 (4%)	10 (37%)	0	2 (7%)
Italy (N = 17)	8 (47%)	3 (18%)	2 (12%)	1 (6%)	0	3 (18%)
Lithuania (N = 16)	2 (13%)	10 (63%)	3 (19%)	1 (6%)	0	0
Poland (N = 19)	8 (42%)	6 (32%)	4 (21%)	1 (5%)	0	0
*Total*	*30 (35%)*	*21 (30%)*	*10 (14%)*	*13 (14%)*	*0*	

Trainings	Community-based organisations/NGOs/Low threshold	Hospitals, clinics or laboratories	Government institutions	Public health institutions	Research or academia	Other
Croatia (N = 11)	6	1		4	0	0
Lithuania (N = 12)	2	10			0	0
*Total*	*8*	*11*		*4*	*0*	*0*

Across the meetings, 78% of evaluation respondents agreed or strongly agreed that there was good representation of all national stakeholders (Table 3). In the evaluation’s free text fields, respondents highlighted the importance of securing high level participation from national government and policy structures and experts from other EU countries.

**Table 3:** Percentage of respondents that agreed/strongly agreed with the evaluation questions

Meetings	Meeting met my expectations	Topics and presentations chosen were appropriate and useful	Presenters were engaging and well prepared	Moderated discussions were useful and relevant	There was a good opportunity to discuss/network during breaks	There was a good representation of national stakeholders	Decisions/action points were made on how to move forward
Croatia (N = 27)	27 (100%)	27 (100%)	27 (100%)	26 (96%)	26 (97%)	22 (82%)	22 (85%)
Italy (N = 17)	14 (82%)	14 (82%)	16 (95%)	15 (77%)	15 (88%)	10 (65%)	10 (67%)
Lithuania (N = 16 or 15)	14 (88%)	14 (93%)	14 (94%)	14 (88%)	16 (100%)	13 (82%)	12 (85%)
Poland (N = 19)	16 (84%)	17 (90%)	17 (79%)	17 (94%)	17 (95%)	16 (84%)	14 (74%)
*Total (%)*	*89%*	*91%*	*92%*	*89%*	*95%*	*78%*	*78%*

Trainings	Training met my expectations	Topics and presentations chosen were relevant for my work	Presenters were engaging and well prepared	Level of discussions and interactions was appropriate	Balance between presentations and group work was appropriate	I gained knowledge which will change my practice
Croatia (N = 11)	10	8	11	10	10	10
Lithuania (N = 12)	12	11	12	12	11	12
*Total (%)*	*96%*	*83%*	*100%*	*96%*	*91%*	*94%*

The situational analyses mapping the national situations against the 2018 ECDC guidance on integrated testing for HIV and viral hepatitis showed that country level policies and practices are not fully aligned with the guidance. A series of structural, professional, financial and other barriers that slow down or block changes towards cross-disease integration were identified: (a) barriers inherent to the health system, e.g. structures separating testing and care for HIV, viral hepatitis, TB and STIs under different mandates; (b) legal and regulatory barriers, e.g. legislation restricting *who* can be offered integrated testing (e.g. only certain patients such as pregnant women and blood donors), or restricting *where* testing can be conducted or *who* may conduct the testing (e.g. not allowing self-testing or trained lay providers to conduct testing); (c) financial constraints or lack of incentives (e.g. lack of reimbursement for HIV testing in primary care or limited financial resources for HIV and STI prevention); (d) lack of national guidelines on how to implement integrated services; and finally (e) national surveillance systems lacking ability to integrate multi-disease data and include data from community testing sites.

The specific topics of interest discussed were: (a) early diagnosis and improved linkage to care for HIV, viral hepatitis and STIs and for the latter two countries TB was also included (Croatia, Poland, Lithuania, Italy); (b) gaps and barriers in current testing policy and practices and how to implement integrated testing (Croatia, Lithuania, Poland); (c) legal barriers for integrated testing and lay-provider testing (Croatia, Poland); (d) cost-effectiveness analysis of different testing strategies (Lithuaia); and finally (e) integration of surveillance data from community testing sites into the national surveillance system (Poland, Italy).

All meetings closed with a plenary session to jointly formulate national level action points and next steps necessary to implement change towards multi-disease approaches. These nationally tailored recommendations on integrated policies and strategies were the main outcomes of the meetings (Table 4).

**Table 4:** Main outcomes: recommendations and next steps

	Italy	Lithuania	Poland	Croatia
Integrated testing policies/strategies	Include monitoring of integrated testing in the national testing policies/strategy		Consolidate current guidelines in terms of HIV/HCV/HBV/STI screening and treatment. Treat rapid testing as a non-medical procedure and indicator to further diagnosis in a medical setting. Move confirmatory tests from community to medical health care setting. HIV should lose its privileged status of the protection of the anonymity of the client. Introduce unique identifier in all testing facilities. Lower legal testing age from 18 to 16	Adapt National Testing Strategy to include new recommendations on integrated testing
Diversification of testing strategies	De-medicalize rapid testing and authorize the administration of rapid testing by lay providers Develop a policy on self-testing	De-medicalize rapid testing and scale-up of testing by GPs	De-medicalize rapid testing (classify as a non-medical procedure). The whole range of combined prevention should be introduced	De-medicalize rapid testing, including self-tests, to provide more accessibility
Stigma and discrimination		Include into the national strategy measures to address stigma in the community and among professionals	Educate doctors on testing (e.g. a guidance on how to offer testing for different STI). There should be a possibility to do the test out of the medical setting (community testing)	Develop tangible solutions including education of GPs/health care staff/medical students and form network of LGBT-friendly GPs
Focus on key populations	Develop national guidelines on Partner Notification	Promote testing uptake among people at high risk and strengthen linkage to care	Establish pathways for confirmatory testing in community testing and for linkage to care	Focus testing on MSM, sex workers and PWID, and minimise loss to care
Cost effectiveness		Conduct analysis of testing strategies	Conduct analysis provide rationale for more testing	Conduct analysis provide rationale for more testing
Surveillance	Develop a uniform way of collecting data from community testing sites	Strengthen documentation, data and cross sector collaborations	Epidemiological data to indicate key populations	
Funding		Secure effective use of existing funds	Secure funds – local level, national and EU funds	Secure funds – local level, national and EU funds
Next steps	Follow-up meeting to be arranged by Ministry of Health to discuss the relevant issues	Follow-up meeting to be arranged by Ministry of Health to start	Cooperation to be strengthened between stakeholders, experts, institutions and government. Inclusion of findings and pilot recommendations into the next edition of the National HIV Programme (in June 2020)	Proposed changes to be presented at the National AIDS Commission meeting

At policy level it was recommended to consolidate existing guidelines and include new recommendations and monitoring of integrated testing in the national testing policies/strategy. For actual cross-disease integration to be feasible, any exceptionality in relation to HIV would have to be abolished and HIV be treated under the same regulations as other infectious diseases. Diversification of testing strategies is a key area requiring action in all countries and recommendations were to de-medicalize rapid testing, scale-up testing by general practitioners and develop policies on self-testing. Another area is stigma and discrimination where in particular action to educate healthcare professionals on multiple disease testing and non-judgmental approaches was highlighted as key. Reaching key populations through increased partner notification, promotion of testing and establishment of easy pathways to improve linkage to care were also recommended.

Across all four meetings (N = 79), respondents to the evaluation survey gave very positive ratings of the agenda, presentations and structure of the meetings, and agreed or strongly agreed that: the meeting meet their expectations (89%), presenters were engaging and well prepared (92%), topics and presentations were appropriate and useful (91%), moderated discussions were useful and relevant (89%), and there was a good opportunity to discuss and network during breaks (95%). While 78% agreed or strongly agreed that decision/action points were made on how to move forward, and 22% neither agreed nor disagreed (Table 3). In the free text fields, respondents listed as best aspects of the meetings the many stakeholders from different institutions and the opportunity for contact between civil society and governmental health institutions, while the mentioned areas for improvement were better involvement of regional representatives and more national stakeholders, equal focus on TB and STIs as opposed to mainly HIV, and more room for discussion.

The meetings were followed-up by activities and actions led by national organisers. For example, in Croatia the organisers agreed to present the recommendations and proposed changes to the National AIDS Commission. In Lithuania the MoH arranged a seminar later in 2019 where key stakeholders met again to continue the discussion of main barriers and how to start implementation of changes. In Poland the meeting served as the final push for a change long underway, namely, to broaden the mandate of the National AIDS Centre to include STI prevention activities, which after an intense process of collaboration with other stakeholders was formalized in August 2019. In Italy, the organisers wrote an official follow-up letter requesting to be invited to the next meeting of the MoH Technical-Scientific Committee to discuss the proposed changes. The request was accepted but the meeting has been postponed due to the COVID-19 crisis.

An overall description of the trainings held in Croatia and Lithuania is provided in Table 1. The trainings were more practice and action-oriented and thus complementary to the policy oriented national multi-stakeholder meetings. Participants were doctors, nurses, social workers and counselors working in clinics, community centers and NGOs (Table 2). Overall, the respondents (N = 23) evaluated the trainings very positively, and agreed or strongly agreed that it met their expectations (96%), topics and presentations were relevant for their work (83%) and presenters were engaging and well prepared (100%) (Table 3). In the free text fields respondents highlighted as very positive the trainers’ approach, the atmosphere of open discussions and gaining new knowledge, while suggestions for improvements included more time overall, e.g. as a weekend course rather than a full day, and more time for discussion in particular, and to conduct trainings in the workplace to allow more staff to take part. It was not possible to assess whether there were additional benefits derived from combining the multi-stakeholder meeting with training, as opposed to having the multi-stakeholder meeting on its own.

## Discussion

The meetings confirmed broad support among national key stakeholders for pushing the cross-disease integration agenda forward. Bringing stakeholders together which may not have regular or any interaction proved to be an effective vehicle for initiating cross-sector discussions and identify actions required for change towards more integrated responses. A strength of the multi-stakeholder meeting format as a tool to foster change, is its adaptability to different national contexts (i.e. the fact that the agenda can be shaped to suit the interests and needs of the country context while still using the same format of situational analysis, discussion, development of recommendations then follow-up). The situational analyses presented at the meetings clearly identified a range of national level barriers, including health and financial silo structures, and legal and regulatory frameworks restricting or impeding internationally recommended service modalities such as lay provider testing. Changing these barriers require political decision-makers to buy-in and actively support the process of diversified and cross-disease testing and care. The meetings confirmed that EU agencies (ECDC, European Monitoring Center for Drugs and Drug Addiction, etc.), international organiations (World Health Organisation, etc.) and expert networks or projects (e.g. European AIDS Clinical Society, Joint Actions and other EU-supported projects) can play a key role in supporting national processes by disseminating guidance and recommendations based on available evidence. Clearly, European level initiatives, guidance and exchange of experiences have the potential to encourage and assist national competent bodies in addressing the above-mentioned barriers and enable improvement in integration across diseases.

A limitation to the situational analysis template used to assess implementation of key interventions (Additional file 1: Annex 1) is that the indicators used are subjective and therefore it will depend upon the presenter what particular issues are raised within the meeting. One aim with the meetings was to bring together relevant national stakeholders to enable cross-disciplinary discussions, but not all who were invited could participate due to competing commitments which was a limitation for the meeting outcomes. In particular, there was a lack of presence from decision-makers, likely due to the low priority of HIV and other infectious diseases on the political agenda, which would be essential to make systems change quickly. While EU supported projects and other international initiatives may be instrumental in setting new agendas and providing ressources for meetings and dialogue activities like the ones described here, the limited time duration makes it hard to generate a sustained long-term push for change. Therefore processes launched at such meetings runs the risk of dying off quickly if local actors are facing difficulties in funding or human resources or do not have strong ties to other stakeholders.

A potential mitigation towards national level system change could be to establish closer dialogue between main stakeholders and the national MoH, with a possible strategy being to create stronger connections to the technical staff at MoH level, instead of aiming for a higher level political one, particularly in scenarios where technical staff can have some influence on what is prioritized at policy level. Clearly, in spite the key stakeholders’ broad support for multi-disease testing and care, changes in health care systems do not come easily. Therefore it is the recommendation of the authors that national level foras are created to increase cross-sectoral dialogue and ensure the continuity of launched intiatives to allow time for change to occur. The continued support of European experts and agencies is of course paramount to foster structural change like cross-disease integration. Poland is an example of this. A first national stakeholder meeting to discuss the country’s HIV response was arranged in October 2016 by the EU supported project OptTEST [8] and a second meeting arranged in June 2019 by INTEGRATE. Action points identified in 2016 as key to an improved response—new testing options in non-medical settings (rapid testing, integrated testing for HIV, HCV, STI) and the need for unique patient identifiers to improve data collection from testing sites—remained unresolved three years later. It was only after sustained national efforts and the EU supported follow-up meeting in 2019 that NAC’s mandate was broadened to include multi-disease testing and prevention activities.

While respecting subsidiarity, EU initiatives may have a key role in fostering cross border cooperation, and exchange of expertise and evidence that may lead to changes at a national level. Clearly, improving communication amongst the partner network, and ensuring political buy, in are essential to utilize all opportunities across Europe to rapidly reduce the pool of undiagnosed persons for HIV, viral hepatitis, TB and STI. National MoHs already have the possibility to request collaboration from EU agencies, and a recommentation is that similar support should be available for other structures to access.

As the focus of the trainings was staff attitudes, communicative skills and interpersonal/cultural competencies, it was key to implement the training as face-to-face sessions to encourage discussion and self-reflection. Moreover, the face-to-face training provided an opportunity to network with other stakeholders involved in similar work and share experiences.

## Conclusions

Through the implementation of national multi-stakeholder meetings as platforms for national level discussion, the Joint Action INTEGRATE has confirmed that in the participating European countries there is great interest and support for integrating HIV, hepatitis, STIs and TB services. However, implementation of actual changes is often slowed or hindered by practical challenges, silo-structures in regulatory, legal and financial frameworks. Continued efforts to further national stakeholder dialogue and cooperation is important to change silo-structures, dismantle barriers and successfully roll-out an integrated approach. EU supported cross-country collaborations, as well as the European Commission’s agencies and institutions, are key arenas and actors when it comes to carving a way forward.

## Supplementary Information

**Additional file 1.Annex 1:** Country Situational Analysis Template.

**Additional file 2.Annex 2:** Meeting Evaluation Form.

## Data Availability

Not applicable.

## Abbreviations

CHIP: Centre of Excellence for Health, Immunity and Infections
CoC: Continuum of care
COVID-19: Corona Virus Induced Disease 2019
CSO: Civil Society Organisation
ECDC: European Centre for Disease Prevention and Control
EMCDDA: European Monitoring Centre for Drugs and Drug Addiction
ETW: European Testing Week
EU: European Union
GP: General Practitioner
HBV: Hepatitis B virus
HCV: Hepatitis C virus
HIV: Human immunodeficiency virus
IC: Indicator Condition
INTEGRATE: Integrating prevention, testing and linkage to care strategies across HIV, viral hepatitis, TB and STIs in Europe
JA: Joint Action
LGBT: Lesbian, Gay, Bisexual & Transgender
MoH: Ministry of Health
MSM: Men who have sex with men
NAC: National AIDS Centre
NGO: Non-Governmental Organisation
PWID: People who inject drugs
STI: Sexually transmitted infection
TB: Tuberculosis
WHO: World Health Organization
