# Supplementary material for: National multi-stakeholder meetings: a tool to support development of integrated policies and practices for testing and prevention of HIV, viral hepatitis, TB and STIs
Source: BMC Infect Dis. 2021 Sep 13;21(Suppl 2):795. doi: 10.1186/s12879-021-06492-y (PMC8436862; doi:10.1186/s12879-021-06492-y)
Supplement: Supplementary file 1 — Additional file 1.Annex 1: Country Situational Analysis Template. [file 12879_2021_6492_MOESM1_ESM.pdf]

Annex 1: Country Situational Analysis Template

**TESTING GUIDANCE**		*[these are subjective – and may depend on the presenters' perspective]*				
**ECDC 2018 – main topics and recommendations**	**What do the National testing Guidelines / policies mention on these topics?**	*What is well implemented in the country?*	*What is partly implemented in the country?*	*What is not implemented?*	*Why is it not implemented?*	**For which topics in national testing guidelines/ policies are updates/changes planned?**
**Combined HIV-Hep C testing**						
**IC-guided testing/ Integrated testing in Healthcare[1] settings**						
**Community testing/ Lay provider testing**						
**Self-testing/ self-sampling**						
**Partner Notification**						
**Re-testing - frequency**						
**Monitoring and Evaluation of testing services**						
Any other key topics specific to your country?						
Combined HIV-STI testing (not in ECDC guidance)						

1 Primary Health Care, Hospital, other healthcare settings (STI clinics, Harm Reduction services, prisons, pharmacies)
