# Supplementary material for: National multi-stakeholder meetings: a tool to support development of integrated policies and practices for testing and prevention of HIV, viral hepatitis, TB and STIs
Source: BMC Infect Dis. 2021 Sep 13;21(Suppl 2):795. doi: 10.1186/s12879-021-06492-y (PMC8436862; doi:10.1186/s12879-021-06492-y)
Supplement: Supplementary file 2 — Additional file 2.Annex 2: Meeting Evaluation Form. [file 12879_2021_6492_MOESM2_ESM.pdf]

Annex 2: Meeting Evaluation Form

**Please state your occupation:**<br>(e.g. Doctor, nurse, social worker, community health worker, government worker, etc.)	
**In which type of setting do you work in?**<br>(e.g. Clinic, hospital, specialty clinic, community centre, non-governmental organisation, etc.)	

Please rate your level of agreement with the following statements regarding the meeting on a 1 to 5 scale; ranging from 1 meaning you strongly disagree with the statement, 3 meaning you neither disagree or agree and 5 meaning you strongly agree.

**National Stakeholder Meeting**	
**Rating**<br>Please circle one number per statement following the grading scale below:<br>1 Strongly disagree<br>2 Disagree<br>3 Neither disagree or agree<br>4 Agree<br>5 Strongly agree	
The meeting met my expectations	1 2 3 4 5
The topics and presentations chosen were appropriate and useful	1 2 3 4 5
The sequence of topics has been well arranged	1 2 3 4 5
The presenters were engaging and well prepared	1 2 3 4 5
The moderated discussions were useful and relevant	1 2 3 4 5
There was a good opportunity to discuss and network during breaks	1 2 3 4 5
There was a good representation of all National stakeholders (NGO's/ clinics/ policy makers/ experts/ etc.) at the meeting	1 2 3 4 5
Decisions/ action points were made on how to move forward	1 2 3 4 5
If a rate between 1-3 has been given, please elaborate further, thanks:	
Were there topics missing from the meeting that you would have liked to see which in your opinion are relevant to the main topic of the meeting?	
What were the best aspects of this meeting?	
What would you do to improve organisation of this meeting?	
Any other comments on the meeting:	
